# Genetic connections and antimicrobial resistance in dogs and owners *Staphylococcus pseudintermedius* isolates

**DOI:** 10.3389/fvets.2025.1652593

**Published:** 2025-12-18

**Authors:** Christina Resende Martins, Roberta Torres de Melo, Clara Mariano Bastos, Rafaela Oliveira Rosa, Amanda Gubert Pereira, Raquelline Figueiredo Braz, Gabriela de Paiva Loures, Belchiolina Beatriz Fonseca, Ana Beatriz Garcez Buiatte, Marcos Bryan Heinemann, Marcus Vinicius Canário Viana, Flávia Figueira Aburjaile, Vasco Ariston de Carvalho Azevedo, Bertram Brenig, Mateus Matiuzzi da Costa, Daise Aparecida Rossi

**Affiliations:** 1 Department of Veterinary Sciences, Universidade Federal de Uberlândia, Uberlândia, Brazil; 2 Graduate Program in Genetics and Biochemistry, Universidade Federal de Uberlândia, Uberlândia, Brazil; 3 Department of Preventive Veterinary Medicine and Animal Health, Universidade de São Paulo, São Paulo, Brazil; 4 Department of Veterinary Sciences, Universidade Federal de Minas Gerais, Belo Horizonte, Brazil; 5 Institute of Veterinary Medicine, Georg-August-Universitat, Göttingen, Germany; 6 Department of Veterinary Sciences, Universidade Federal do Vale do São Francisco, Petrolina, Brazil

**Keywords:** *mecA*, *msrA*, *fnbB*, Panton-valentine leukocidin, resistance

## Abstract

The study’s objective was to isolate *Staphylococcus pseudintermedius* from dogs with superficial pyoderma and/or recurrent otitis, and their guardians, to determine oxacillin, cefovecin, and gentamicin resistance, associated risk factors for infection, and genetic similarity between isolates from dogs and guardians. Prevalence of *S. pseudintermedius* in dogs was 76% and in humans was 56%, with concurrent identification in 44%. Oxacillin resistance occurred in 6.38% of dog isolates and 11.36% of isolates from guardians, with relatively strong (disk diffusion test) or moderate (Minimum Inhibitory Concentration) association between phenotypic testing and *mecA* gene presence. For cefovecin, dog isolates presented resistance in 8.15% disk diffusion and 23.40% broth microdilution. In humans, 6.81 and 36.36% showed cefovecin resistance in disk diffusion and Minimum Inhibitory Concentration tests. Gentamicin resistance in dogs was identified in broth microdilution testing in 2.12%. *fnbB* was identified in 4.39% of isolates with relatively strong association between results of dogs and humans. Dogs sleeping with *S. pseudintermedius*-positive humans were 6 times more likely to test positive, and dogs attending grooming sessions were 4 times more likely positive for *S. pseudintermedius*. The whole genome sequencing revealed transmission between dogs and humans in 3 cases. Resistance genes for 8 antibiotic classes were found in chromosomal and plasmid genomes.

## 1 Introduction

*Staphylococcus pseudintermedius* (SP), along with two other species, *S. intermedius* and *S. delphini*, both coagulase-positive, form the group known as the *Staphylococcus intermedius* group (SIG) (1). Like *S. aureus*, SP can produce a variety of virulence factors, including coagulase, thermonuclease, proteases, surface proteins such as clumping factors and protein A, as well as cytotoxins, exfoliative toxins, and enterotoxins (2).

SP is the most frequently isolated species in cases of pyoderma in dogs, often identified in ears and infected wounds, and can complicate immunomodulatory responses (3, 4). Another critical aspect regarding SP is its ability to develop resistance to multiple drugs. In this context, Methicillin-resistant *Staphylococcus pseudintermedius* (MRSP), characterized by the presence of the *mecA* gene, has emerged as a significant challenge for small animal veterinarians, especially veterinary dermatologists, due to its extensive drug resistance and capacity to cause nosocomial infections (5). MRSP is increasingly isolated from dogs and has also been identified as a causative agent of infections in humans (6–9).

Other genes are associated with antimicrobial resistance in *Staphylococcus* species and may complicate treatment in cases of infection, such as the *msr(A)* gene, which encodes a macrolide efflux protein (10). Additionally, other mechanisms may be involved in *β*-lactam resistance, such as the production of β-lactamases. In this context, the *blaZ* gene, whose expression is regulated by the *blaI* and *blaR1* genes, is frequently reported (11–13).

Regarding its zoonotic potential, reports of MRSP infections in humans are increasing worldwide, with isolates found in wounds from bites or other skin infections, as well as external otitis, sinusitis, catheter-related bacteremia, pneumonia, and brain abscess (14–16). Furthermore, it has been demonstrated that guardians of dogs with deep pyoderma are often carriers of SP (17), and studies suggest transmission of MRSP from dogs to their guardians (18, 19) and to veterinary hospital employees (20).

The whole genome sequencing (WGS) has been employed in the investigation of SP in animals and humans, contributing to the characterization of virulent strains and aiding in the tracking of multidrug-resistant strains (21, 22). However, this technology is not universally available, as countries with lower and middle development have limitations in accessing such tools. Few studies in Brazil have utilized these technologies in the investigation of SP in dogs (23, 24). Therefore, the objective was to isolate, characterize and correlate risk factors for infection of SP and associate the isolates from dogs with superficial pyoderma and/or recurrent otitis and their guardians.

## 2 Methods

### 2.1 Samples from the dogs and their guardians

A cross-sectional study was conducted in which clinical samples from 50 animals (Supplementary Table S0) with otitis and/or pyoderma treated at public and private veterinary hospitals, as well as from their guardians, were evaluated to determine the simultaneous presence of positivity, hypothetical risk factors, and prevalence. The criteria for the sample size were limited to the time available for data collection within the study.

The inclusion criteria for the study were dogs that had recurrent superficial otitis and/or pyoderma on at least three separate occasions, regardless of previous use of topical and/or systemic antimicrobial medications. As an exclusion criterion, only animals that had not received antibiotics in the previous 15 days were selected. Animals and/or humans who were using antimicrobials at the time of clinical evaluation were excluded from the study.

For the identification of superficial pyoderma lesions, we considered the presence of circular alopecia, papules, pustules, erythema, and epidermal collarettes. To characterize otitis, we considered the presence of otological itching or pain, as well as erythema, edema, and any type of ear discharge.

The guardians also responded to a questionnaire regarding the dogs’ lifestyle habits, interactions with humans, progression of the animals’ disease, and treatments already administered (Supplementary Table S1).

Material from the external ears was collected using a veterinary otoscope and sterile swabs, which were then stored in Stuart medium (Absorve®). Skin samples were collected similarly, preferably from intact pustules (previously punctured with a hypodermic needle) or epidermal collarettes at the lower portion of vesicobullous or pustular lesions. Nasal samples from guardians were self-collected using swabs under research supervision. Following collection, samples were immediately transported to the laboratory for culture and identification.

This study was approved by the Animal Ethics Committee (CEUA) under protocol number 055/18, and by the Human Research Ethics Committee (CEP) under protocol number 2,980,894.

### 2.2 Culture and identification of *S. pseudintermedius* isolates

The samples were enriched in Brain Heart Infusion broth (Oxoid®) for 12 h at 37 °C and subsequently streaked onto Baird-Parker agar (Oxoid®) with incubation at 37 °C for 24 h. Three colonies showing typical black appearance, with or without a halo indicative of lecithinase activity, were subcultured onto Baird-Parker agar (Oxoid®) and further incubated at 37 °C for 24 h. Pure colonies were phenotypically tested for coagulase and catalase enzyme production and morphologically identified by Gram staining (25). Colonies displaying phenotypic characteristics of coagulase-positive *Staphylococcus* were genotypically analyzed using PCR for SP identification, and simultaneously preserved in UHT milk (Ninho®, Nestlé) at −80 °C in an ultrafreezer.

DNA extraction was performed using a heat-based method. A loopful of colonies grown on Baird-Parker agar (Oxoid®) was transferred into 100 μL of TE buffer (10 mM Tris HCl, 5 mM EDTA, pH 8.0) in a 1.5 mL microcentrifuge tube. The mixture was heated at 90 °C for 15 min to release DNA into the buffer. The tube was then frozen completely for approximately 30 min and thawed at room temperature. After thawing, the mixture was vortexed to homogenize and centrifuged at 14,000 rpm for 5 min. The supernatant containing the DNA was carefully collected using a 20 μL pipette and stored at −20 °C.

For the identification of SP, primers previously described by Sasaki et al. (26) targeting the *nuc* gene were used (Forward primer: F2 TRGGCAGTAGGATTCGTTAA; Reverse primer: R5 CTTTTGTGCTYCMTTTTGG). The PCR reactions were prepared using 12.5 μL of GoTaq® Green Master Mix, 2.5 μL of each primer (10 pmol/μL), 7 μL of GoTaq® Green water, and 3 μL of total DNA. The amplification protocol included an initial denaturation cycle at 95 °C for 2 min, followed by 35 cycles of denaturation at 95 °C for 30 s, annealing at 56 °C for 35 s, and extension at 72 °C for 1 min. A final extension step was performed at 72 °C for 2 min.

Subsequently, agarose gel electrophoresis was conducted using a 1.5% agarose gel with a 100 bp molecular weight marker at 80 V, 80 mA, and 100 W for 120 min (Thermo Scientific®). After gel electrophoresis, gel imaging was performed using a UV transilluminator (L-PIX EX®). Presence of a band at 926 base pairs (926 bp) indicated the sample as SP (26).

The MRSP 3279 strain of SP served as positive control, while an amplification reaction without added DNA served as a negative control. We selected only samples where SP was identified in both dogs and humans for analysis.

### 2.3 Virulence and antimicrobial specific genes

The same previously extracted DNA was used, and amplifications were carried out using 12.5 μL of GoTaq® Green Master Mix, 2.5 μL of each primer for the respective genes (10 pmol/μL) (Table 1), 7 μL of GoTaq® Green water, and 3 μL of total DNA.

**Table 1:** Sequence and base pairs of the genes investigated in the isolates of SP from dogs and their guardians.

Gene	Sequence (5′ → 3′)	Molecular weight	Reference
*mec*A	F (5′- F ACTGCTATCCACCCTCAAC-3′) R (5′- CTGGTGAAGTTGTAATCTGG- 3′)	100	(24)
*mec*A1	F (5′-TCCAGATTACAACTTCACCAGG-3′) R (5′-CCACTTCATATCTTGTAACG-3′)	162	(25)
*mec*A2	F (5′-ATCGATGGTAAAGGTTGGC-3′) R (5′-AGTTCTGCAGTACCGGATTTGC-3′)	540	(25)
*msr*A	F (5′-TCCAATCATTGCACAAAATC-3′) R (5′-AATTCCCTCTATTTGGTGGT-3′)	163	(26)
*fnb*B	F (5′-GGAGCGGCCTCAGTATTCTT-3′) R (5′′-AGTTGATGTCGCGCTGTATG-3″)	201	(27)
PVL	F (5′ – GCTGGACAAAACTTCTTGGAATAT – 3′) R (5′ – GATAGGACACCAATAAATTCTGGATTG – 3′)	85	(28)

The amplification was conducted under the following conditions: an initial denaturation cycle at 95 °C for 5 min; followed by 35 amplification cycles (denaturation at 94 °C for 2 min, annealing at 53 °C for 2 min, and extension at 72 °C for 1 min); and a final extension at 72 °C for 7 min. After gel electrophoresis, gel imaging was performed using a UV transilluminator (L-PIX EX®). The presence of each gene was determined by the size of the bands in base pairs as per Table 1. The MRSP 3279 strain of SP was used as positive control, and a reaction without added DNA served as a negative control.

### 2.4 Antimicrobial susceptibility (*disk diffusion test*)

SP (from dogs and humans) were thawed and reactivated in Brain Heart Infusion broth (Oxoid®) for 12 h at 37 °C. Subsequently, they were streaked onto Tryptic Soy Agar (TSA, BD®) plates and incubated for 24 h at 37 °C. Three colonies were selected using a sterile loop, suspended in sterile 0.9% NaCl solution until reaching turbidity equivalent to 0.5 McFarland standard (27), and then streaked onto Muller-Hinton agar (Oxoid®).

After the agar drying, antimicrobial disks containing oxacillin (1 μg – Cefar®), gentamicin (10 μg – Cefar®), and cefovecin (30 μg – Oxoid®) were applied. After 18 h of incubation at 35 °C, the inhibition zones were measured and compared against reference values provided by CLSI (27). The resistance zone diameter breakpoints for SP were defined as: gentamicin ≤12, oxacillin ≤17, and cefovecin ≤20 (Table 2).

**Table 2:** Indexes obtained from disk diffusion testing compared to the gold standard (MIC).

	Oxacilin	Cefovecin	Gentamicin
Susceptibility	0.3784	0.2222	0.000
Specificity	0.9259	0.9844	0.9623
Positive predictive value	0.7778	0.8571	0.000
Negative predictive value	0.6849	0.7500	0.9273
Likelihood ratio	5.108	14.220	0.000
*p* value	0.0009	0.0025	0.9999

### 2.5 Minimum inhibitory concentration

The same reactivated and adjusted cultures used for the disk diffusion test were employed for the determination of Minimum Inhibitory Concentration (MIC), using the antimicrobials gentamicin (Novafarma®), oxacillin (Novafarma®), and cefovecin (Convenia®, Zoetis). Microdilution method was used in Mueller-Hinton broth (Oxoid®) adjusted with 0.37 g/100 mL of Ca^2+^ and 0.835 g/100 mL of Mg^2+^ (27). Concentrations tested were 0.25, 0.5, 1, 2, 4, 8, 16, 32 μg/mL for the different antibiotics. The resistance breakpoints for SP were defined as: gentamicin ≥16, oxacillin ≥0.5, and cefovecin ≥2 (27, 28).

For analysis, 180 μL of adjusted MH broth (Oxoid®) was added into 96-well microdilution plates containing the target antimicrobials at previously established concentrations. Subsequently, 20 μL of adjusted bacterial suspension was added (28). The plates were then incubated at 35 °C for 18 h for gentamicin and cefovecin samples, and for 24 h for oxacillin (27, 28). After incubation, visual reading was conducted, defining MIC as the lowest antibiotic concentration at which no visible bacterial growth was observed, indicated by the absence of turbidity in the medium (Table 2).

### 2.6 DNA extraction and sequencing

The DNA was extracted using CTAB (cetyltrimethylammonium bromide) buffer (Sigma-Aldrich). Colonies of SP grown in one petri plate were resuspended in 10 mL of PBS. Then centrifuged at 12.000 g, at 25 °C for 20 min. A total of 500uL of CTAB buffer (100 mM Tris–HCl pH 8.0. 20 mM sodium EDTA pH 8.0; 1.4 M NaCl; 2% CTAB) and 10uL of proteinase K was added in the pellet which was incubated at 65 °C per 60 min and homogenized every 10 min. After, the sample was centrifuged at 12.000 g, at 25 °C for 10 min. The pellet was discarded, and 500uL of a solution chloroform/isoamyl alcohol (24v:1v) was added, homogenized and incubated for 5 min at 25 °C. The supernatant was centrifuged at 6000 g for 10 min at 25 °C and cold isopropanol added (0.65x the volume of supernatant). Then it was incubated at −20 °C for 1 h and centrifuged at 12.000 g for 30 min at 25 °C. The supernatant was discarded, the pellets washed with cold ethanol 70% and centrifuged at 12000 g for 10 min at 25 °C. Finally, the supernatant was dry, eluted in water and lyophilized until the use.

A 450 bp read library was constructed using NEBNext Fast DNA Fragmentation and Library Preparation Kit (New England Biolabs, Ipswich, NE, United States) and quality checked by the Agilent 2,100 Bioanalyzer. The gDNA library was then sequenced by Illumina HiSeq 2,500 sequencing platform (Illumina, San Diego, CA, United States), 2 × 150 bp paired end, insert size 500 bp. Read quality was assessed using FastQC (29) and read trimming was performed using Fastp v. 0.23.4 (30).

### 2.7 Genome assembly, annotation, pan-genome and phylogenomic analysis

The genomes were assembled using SPAdes v. 3.15.5 (31). Contigs with sizes smaller than 200 bp were removed using Seqkit v. 2.8.0 (32). Completeness and contamination were evaluated using CheckM2 v. 1.0.2 (33) while fragmentation was evaluated using QUAST v. 5.0.2 (34). Contaminant contigs were removed using BV-BRC’s MetagenomicBinning (35). Taxonomy was checked using GTDB-Tk v. 2.3.2 with database r220 (36) and comparison against the reference genome *S. pseudintermedius* SP_11304-3A (GCA_016126715.1), using a threshold of >95% Average Nucleotide Identity (ANI) calculated with FastANI v. 1.32 (37). Additionally, genome-wide ANI were computed for all pairwise comparisons of genomes using FastANI.

Genome annotation was performed using Prokka v. 1.14.6 (38). To characterize the entirety of genes in the dataset (pan-genome), Panaroo v. 1.2.7 (39) was employed with the strict option to ensure inclusion of only high-quality genes. Nucleotide sequences of individual gene families were aligned using Prank (40). Core genes were defined as those present in ≥ 99% of the genomes, while accessory genes were those present in ≥ 0% and < 99% of the genomes. Single Nucleotide Polymorphisms (SNPs) were extracted from the alignments generated by Panaroo using snp-sites v. 2.5.1 (41). Subsequently, a maximum likelihood phylogenetic tree using RAxML v. 8.2.12 (42) was constructed with a general time reversible (GTR) model (43) and a Gamma model of nucleotide substitution. The phylogenetic tree was visualized using the Interactive Tree of Life (iTOL) online platform (44). For pairwise comparison of genomes, genetic distances were calculated based on SNPs from the concatenated alignment of core genes using snp-dists v. 0.8.2.^1^

### 2.8 *In silico* identification of sequence type, antimicrobial resistance and virulence genes, and plasmid reconstruction

The sequence types (ST) of each isolate were determined using MLST v.2.19.0,^2^ which extracts seven single copy housekeeping genes (*ack, cpn60, fdh, pta, pura, sar* and *tuf*) and compares their sequence identity to previously deposited allele combinations in the *S. pseudintermedius* PubMLST database.^3^ All newly identified Sequence Types (STs) were submitted to pubMLST (Supplementary Table S3).

ABRicate v.1.0.0^4^ was used to determine the presence of virulence genes using threshold values of >80% sequence identity and >80% sequence coverage. Virulence genes from the isolates were compared to those found on the Virulence Factor Database (VFDB) (45). AMRFinderPlus (46) was also used to determine the presence of antibiotic and biocide resistance genes, using threshold values of >80% sequence identity and >80% sequence coverage.

MOB-suite v3.1.4 (47) was used to predict plasmid sequences from the assembly genome and identify their replicon types and mobility. AMRfinderPlus (46) was then employed to identify antibiotic and biocide resistance genes in the plasmid sequences, with threshold values of >80% sequence identity and >80% sequence coverage.

### 2.9 Statistical analysis

The data was tabulated and subjected to descriptive statistics. Calculations were performed using GraphPad Prism 8.3. To assess the association between positivity in dogs and their guardians and the influence of risk factors “bathing location” and “sleeping location of the animal, the Fisher’s exact (*p* < 0.05) was used, followed by calculation of the Odds Ratio (OR). Comparisons related to gender, age, and race were made using Fisher’s test (*p* < 0.05) (48).

To determine if there was an association between the presence of resistance and virulence genes in dogs and humans, the Phi (ϕ) coefficient test (*p* < 0.05) was employed. The strength of association was classified according the ϕ coefficient value (49, 50): very strong (0.8–1), strong (0.6–0.8), relatively strong (0.4–0.6), moderate (0.2–0.4), weak (0.1–0.2), insignificant or nonexistent (0–0.1) (49).

The association between susceptibility results for oxacillin and cefovecin by disk diffusion method and Minimum Inhibitory Concentration (gold standard) was evaluated using the Fisher’s exact test (*p* < 0.05). Susceptibility, specificity, positive predictive value, negative predictive value, and likelihood odds were also calculated (48).

All 21 sequenced strains had negative results for antimicrobial resistance using the disk diffusion method. The concordance between genotypic and phenotypic resistance, as determined by MIC, was assessed for oxacillin, cefovecin, and gentamicin. For the evaluation of diagnostic performance, phenotypic resistance was considered the gold standard, with genotypic results being compared to this reference.

Associations were evaluated using Fisher’s exact test (two-tailed, *p* < 0.05) for 21 sequenced strains. Diagnostic performance metrics included susceptibility, specificity, PPV, NPV, Odds Ratio, true positives and true negatives. For gentamicin, all three aminoglycoside resistance genes (*aac(6′)-Ie/aph(2″)-Ia, ANT(6)-Ia/aad(6),* and *aph(3′)-IIIa*) were analyzed independently, while *β*-lactam resistance was evaluated through the complete *blaIZR1* operon. Statistical analyses were performed using Rstudio 2024.0.9.1 (51) with the tidyverse (52) epiR (53) and caret (54) packages.

### 2.10 Ata availability

The genomes of the 21 SPs are isolated from dogs and their guardians that have been sequenced are available on the National Center for Biotechnology Information (NCBI) database, bioproject PRJNA1135159 (Supplementary Table S3).

## 3 Results

### 3.1 Distribution of sample profiles according to descriptive and microbiological characteristics

The animals were grouped according to characteristics related to isolated strains, antimicrobial sensitivity, and the presence of resistance genes. Considering the 50 animals used in the study, females accounted for 26/50 (52%) and males for 24/50 (48%) of the sample. The most predominant breed was Shih Tzu 17/50 (*p* = 0.0338 - Fisher’s exact test), followed by mixed-breed 7/50 (14%), Bulldog 5/50 (10%), Pug 4/50 (8%), Golden 4/50 (8%), Maltese 4/50 (8%), Yorkshire 3/50 (6%), Cocker Spaniel 2/50 (4%), and others 4/50 (8%). The age range was 1 to 5 years 32/50 (*p* = 0.0012 - Fisher’s exact test), followed by 6 to 10 years 15/50 (30%) and 11 to 15 years 3/50 (6%) (Supplementary Table S0).

### 3.2 The occurrence of SP in dogs and their guardians was high, and the resistance levels varied according to the phenotypic and genotypic methods employed

The 100 clinical samples from dogs and their guardians yielded a total of 289 colonies, which were tested for the presence of SP, with one sample from each owner’s nostril, one skin and/or ear sample from each dog, and two samples from animals with simultaneous otitis and pyoderma, with three colonies selected per animal sample. Among colonies from dogs and humans, 96/153 (62.94%) and 96/136 (70.59%), respectively, tested positive for SP. The prevalence of SP isolates in dogs was 38/50 (76.00%) and in humans it was 28/50 (56%). Simultaneous identification in samples from dogs and their guardians occurred in 22/50 (44.00%) of cases. There was a positive association between the presence of SP in the dog and in its owner, with humans having five times greater odds of acquiring this bacterium when in contact with a positive dog (*p* < 0.025, OR = 5).

From the 22 dogs positive for SP on the skin or ear, 47 strains were obtained, among which 11 (23.40%) were resistant to oxacillin by disk diffusion testing (Supplementary Table S2). Among these, 3/11 (27.27%) harbored the *mecA* gene. Using MIC, 14/47 (29.78%) were resistant to oxacillin, and of these, 4/14 (28.57%) strains were positive for the *mecA* gene.

Eight out of the 11 strains (72.72%) showing oxacillin resistance in the disk diffusion test and 10/14 (71.42%) in MIC did not carry the *mecA* gene. Conversely, 2/36 (5.56%) samples susceptibility to oxacillin in the disk test and 1/33 (3.03%) in MIC were positive for the *mecA* gene.

Among the 44 strains isolated from 22 tutors, 7 (15.90%) were resistant to oxacillin in the disk diffusion test, with 3/7 (42.85%) positive for the *mecA* gene. In MIC, 23/44 (52.27%) were resistant, and 5/23 (21.74%) carried the *mecA* gene. Among the resistant strains, four in the disk diffusion test and 17 in MIC were negative for the *mecA* gene. Meanwhile, 2/44 (4.5%) strains susceptible to oxacillin in both *in vitro* tests were positive for the *mecA* gene.

Only 5/47 (10.64%) SP strains isolated from dogs were positive for any *mecA* gene, with two strains positive for *mecA* and *mecA1* genes, two for *mecA*, *mecA1*, and *mecA2* genes, and one positive for the *mecA2* gene. From the tutor-isolated strains, 5/44 (11.36%) were positive for any *mecA* gene, with three positives for *mecA* and *mecA1* genes, one for *mecA*, *mecA1*, and *mecA2* genes, and one for the *mecA1* gene. In two strains susceptibility in disk diffusion and resistant in MIC, the *mecA* gene was identified.

Among SP strains isolated from dogs, 3/47 (6.38%) showed resistance to cefovecin in the disk diffusion test and 9/47 (19.14%) in MIC. In the 44 tutor-isolated strains, 3 (6.81%) and 16 (36.36%) strains were resistant in the disk diffusion and MIC tests, respectively.

For oxacillin-resistant strains in phenotypic tests (set of isolates from dogs and guardians), there was a relatively strong association between oxacillin resistance in the disk diffusion test and the presence of the *mecA* gene (ϕ coefficient = 0.430), and this association was moderate for MIC (ϕ coefficient = 0.294). In other words, *in vitro* oxacillin resistance was not an important or sole parameter for identifying the *mecA* gene.

In 42/47 (89.36%) dog isolates, previous use of some antimicrobial was reported, with 29/42 (69.04%) belonging to the *β*-lactam class. There was a strong association between previous use of β-lactam antimicrobials and resistance in both disk susceptibility testing (ϕ coefficient 0.682) and MIC (ϕ coefficient = 0.694) for oxacillin.

None of the SP isolates from dogs were resistant to gentamicin in the disk diffusion test; however, in the MIC test, 1/47 (2.12%) demonstrated resistance. Among the isolates from the humans, 2/44 (4.55%) showed resistance to gentamicin in the disk diffusion test, and 4/44 (9.09%) in the MIC (Supplementary Table S2). There was no association between the MIC values of SP isolated from dogs and their tutors for gentamicin.

The *msrA* gene was identified in 2/47 (4.25%) of the dog isolates and in 2/44 (4.54%) of the tutor isolates. There was no association between the presence of the *msrA* genes in dogs and their guardians.

The indices obtained from the disk diffusion test when compared to the gold standard (MIC) are described in Table 2.

### 3.3 The occurrence of the *fnbB* gene was low, and no samples exhibited gene related to Panton-valentine leucocidin (PVL) production

The *fnbB* gene was identified in only 4/91 (4.39%) isolates, with 3 (75%) from dogs and 1 (25%) from a tutor, showing relatively strong association between the presence of this gene in isolates from dogs and tutors (ϕ coefficient = 0.563). The PVL-producing gene was not identified in any SP isolates from samples of dogs or their tutors.

### 3.4 There was a low association between lifestyle habits and the presence of SP in dogs and their tutors

The association between where the dog sleeps (whether with the owner or in its own bed) and the isolation of SP in dogs and tutors did not represent a risk factor for colonization in humans (*p* = 0.3869, OR = 1.857). However, we observed that dogs that sleep with SP-positive humans are six times more likely to become SP-positive themselves compared to those sleeping in their own bed (*p* = 0.0264, OR = 6).

Regarding the location where the dog is bathed, we observed that this is not a factor influencing SP positivity in tutors (*p* = 0.7734, OR = 0.7618). However, despite the association not being statistically significant (*p* = 0.0519), the *p* value showed a tendence, and dogs bathed at grooming salons have a four-fold higher chance (OR = 4.091) of being colonized by SP compared to those bathed at home.

### 3.5 WGS showed a diverse population, with similarity in some SP genomes isolated from dogs and their guardians

**Figure 1:**
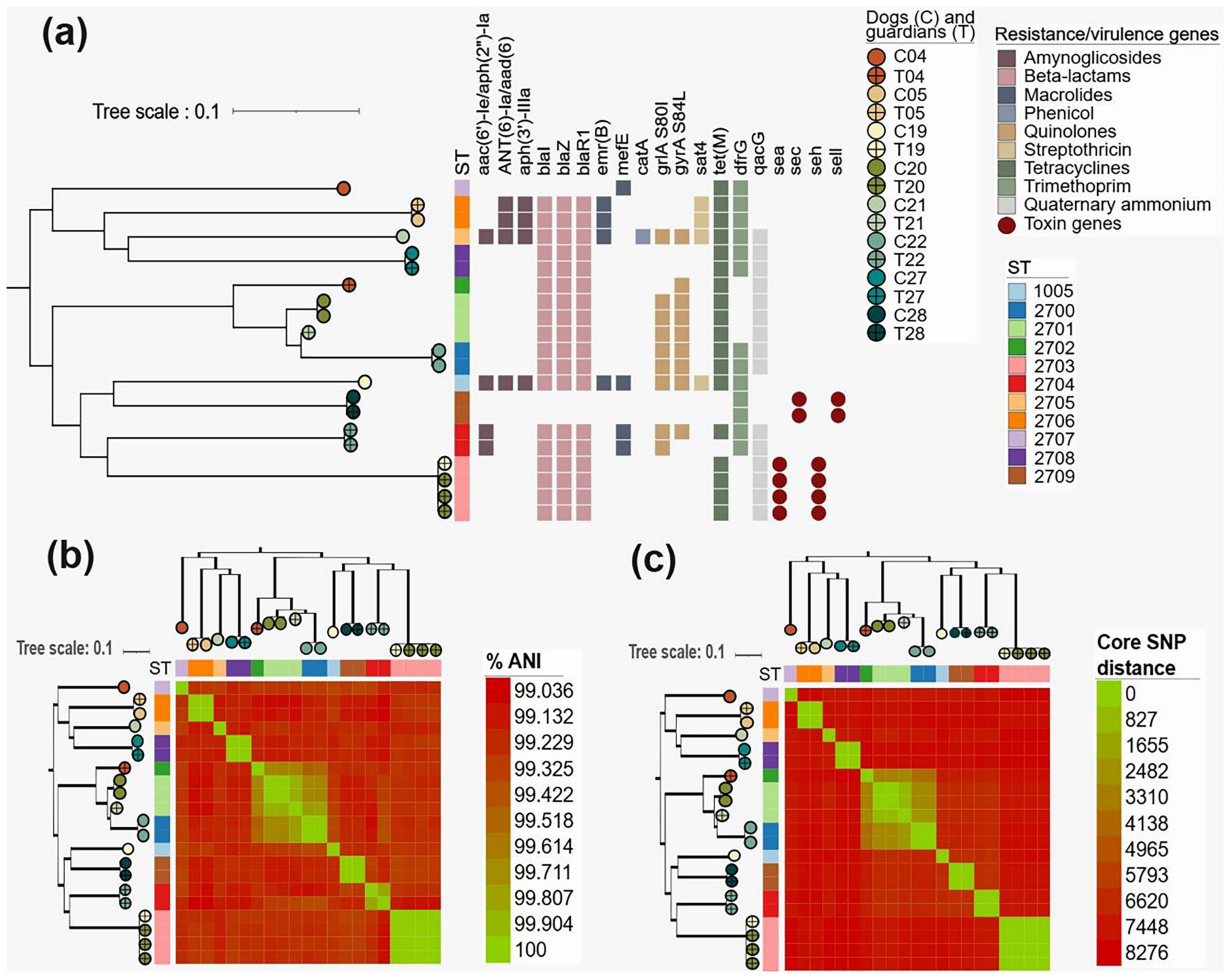
Genomic relationships, ANI, and SNP distances among *Staphylococcus pseudintermedius* isolates from dogs and their guardians. **(a)** Core-genome phylogeny of *S. pseudintermedius* isolates showing sequence types (STs) and the presence/absence of antimicrobial resistance (AMR) and virulence genes. Colors indicate host source (dogs, guardians) and STs. **(b)** Heatmap of average nucleotide identity (ANI, %) among isolates, with dendrogram clustering to highlight genomic similarity within and between hosts. **(c)** Core single nucleotide polymorphism (SNP) distance matrix with hierarchical clustering, illustrating genetic diversity and potential host-sharing relationships.

Strains were kept frozen until DNA extraction for WGS. When we reactivated the strains, only 21 were viable for DNA extraction. Then, we sequenced the genomes of 21 SP isolates collected from humans (11/21–52.38%) and their dogs (10/21–47.62%). The genomes contained between 23 and 186 contigs, N50 values range between 45,986 and 590,378 bp, and the sequences sizes range from 2.47 to 2.96 Mb (mean 2.60 Mb) (Supplementary Table S3). The ANI values of each genome compared to the reference genome ranged from 98.91 to 99.27%. We identified 3,351 genes families in the pangenome of all SP genomes. A total of 2,045/3,351(61.03%) genes comprised the core genome (present in ≥ 99% genomes), and the number of accessory genes per genome ranged from 233 to 826. To examine the phylogenetic relationships of the isolates, we constructed a maximum likelihood tree based on SNPs extracted from nucleotide alignments of 2,045 core genes (Figure 1a).

Multi-locus sequence typing (MLST) analysis allowed us to identify only 1 STs previously described (C019B), ST 1005. We identified 10 new STs, which were submitted to pubMLST database (Supplementary Table S3; Figure 1a).

We identified resistance genes to 8 classes of antibiotics and 1 heavy metal class (Supplementary Table S3; Figure 1a). One genome isolated from a dog showed at least one resistance gene related to all classes (C21), and no genome was negative for all genes. The most frequent genes were those characterizing resistance to beta lactams, *blaI, blaR1* and *blaZ*, and tetracyclines, *tetM*, which were present in 18/21 (85.71%) of the samples. Resistance to trimethoprim, defined by the *dfrG* gene, was the fourth most frequent, present in 13/21 (61.90%) of the samples. About quinolones, two points of mutations were found in the *grlA* (S80I) and *gyrA* (S84L) genes, both in 9/21 (42.86%) of the genomes. Resistance to aminoglycosides was defined by the genes *aac(6′)-Ie-aph(2″)-Ia* (4/21–19.05%), *aph(3′)-IIIa* (4/21–19.05%) and *anti(6)-Ia/aad(6)* (4/21–19.05%). Regarding macrolide resistance, the *mefE* (4/21–19.05%) and *ermB* (4/21–19.05%) genes were found. The *sat4* gene, related to streptothricin resistance, was identified in 4/21 (19.05%) of the samples. One SP genome (4.76%) isolated from a dog had the *catA* gene, which is related to resistance to phenocols. Finally, *qacG* gene, related to resistance to quaternary ammonium compounds, was present in 15/21 (71.43%) genomes. The *mecA* gene was not identified in the genomes by AMRfinderplus, and it was also not detected by PCR in the sequenced samples. The *msrA* gene was also not found in the genomes, but it was identified by PCR in one guardian SP genome (T019).

The analysis of the relationship between resistance genes and phenotypic profiles (Supplementary Table S4) demonstrated that the *aac(6′)-Ie/aph(2″)-Ia* gene showed modest performance in predicting gentamicin resistance, with a susceptibility of 33.3%, specificity of 83.3%, and an odds ratio of 2.37, although without statistical significance. In contrast, the *ANT(6)-Ia/aad(6) and aph(3′)-IIIa* genes showed no association with the resistant phenotype (susceptibility = 0%). For oxacillin and cefovecin, the *blaIZR1* operon exhibited 100% susceptibility, but very low specificity (<20%), resulting in a high rate of false positives, which significantly limits its positive predictive value (PPV < 30%). No statistical significance was observed.

**Figure 2:**
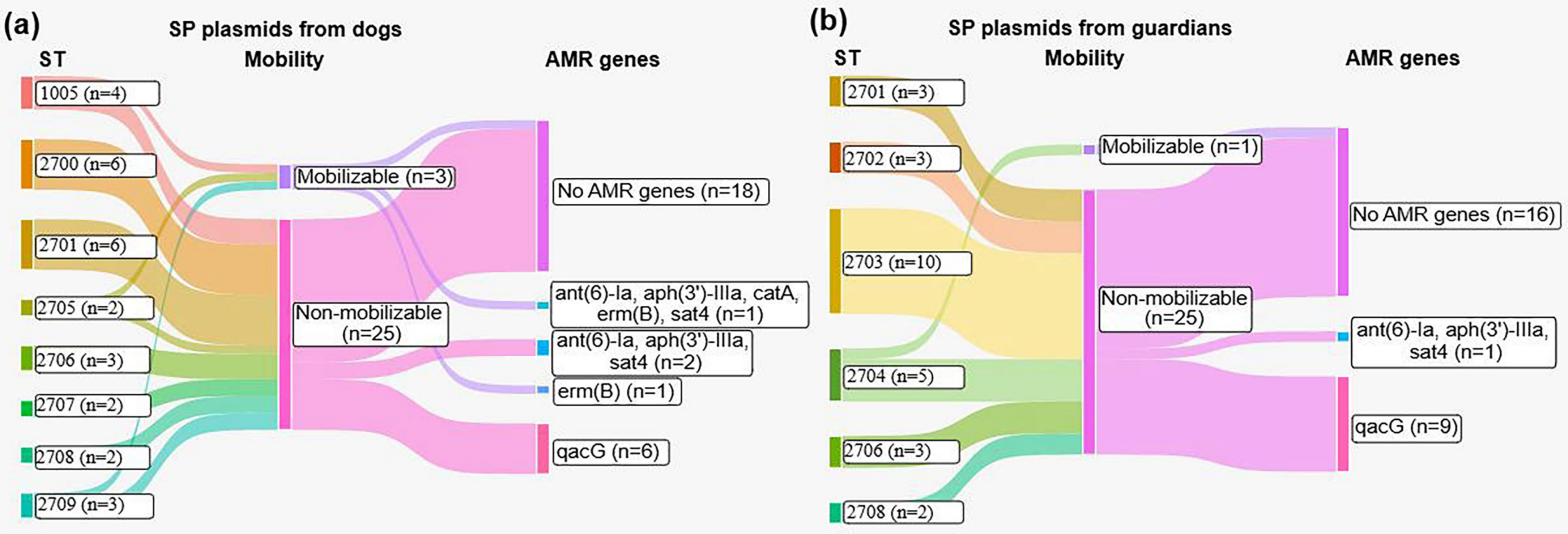
Mobility and antimicrobial resistance genes of *S. pseudintermedius* plasmids from dogs and their guardians. **(a)** Sankey diagram showing distribution of plasmid mobility and AMR genes among plasmids detected in *S. pseudintermedius* isolates from dogs. **(b)** Sankey diagram showing plasmid mobility and AMR gene profiles among plasmids identified in isolates from dog guardians. The width of the flows is proportional to the number of plasmids in each category.

We utilized the mob-suite (47) to identify plasmids, followed by verification of the presence of antibiotic and biocide resistance genes in the plasmid sequences using AMRfinderPlus (46). A total of 54 plasmids were identified, with 28/54 (51.85%) found in 10/10 (100.00%) genomes of dog-associated SP and 26/54 (48.15%) in 10/11 (90.90%) genomes of guardian-associated SP (Supplementary Table S5). We identified 4/54 (7.41%) mobilizable plasmids, of which 3/4 (75.00%) were from dogs and 1/4 (25.00%) from humans, and 50/54 (92.59%) non-mobilizable plasmids, evenly distributed between dogs and humans SP (Figures 2a,b). The plasmids were present across all STs, with between 0 and 5 resistance genes identified per plasmid. One dog SP plasmid (C21B, Supplementary Table S5) carried five resistance genes (*ant(6)-Ia, aph(3′)-IIIa, catA, erm(B), sat4*). Two dog SP plasmids and one human SP plasmid carried 3 resistance genes (*ant(6)-Ia, aph(3′)-IIIa, sat4*). One dog plasmid harbored the *erm(B)* gene, which was absent in human plasmids. The *qacG* gene was found in 9 human and 6 dog SP plasmids.

As for virulence, 4/21 (19.05%) genomes had the *sea and seh* genes, all of them isolated from humans. Two (9.52%) had *sec* and *sell* genes, isolated from a dog and his owner. The *pvl* and *fnbB* genes were not found by VFDB, which coincided with the PCR results.

Using the ANI values and core SNP distance, we constructed matrices to show the similarity between the samples (Figures 1b,c). Genome-wide ANI values for every possible pair of SP genomes ranged from 99.04 to 100.00% (Figure 1b; Supplementary Table S4). Core SNP distance for every possible pair of genomes ranged from 0 to 8,276 (Figure 1c). We observed greater similarity between samples of the dog and its owner in three cases (T05 and C05, T027 and C027, and T028 and C028). For the other genomes, we observed high similarity between samples isolated from the same owner (T022A and T022B, T020A, T020B and T020C) or from the same dog (C020A and C020C, C022A and C022B). We also observed a high similarity between the guardian genomes T019B T020, which were clustered together and showed the same resistance and virulence profile.

## 4 Discussion

The prevalence of SP in dog samples varies; however, most studies show percentages between 80 and 91% positivity for this pathogen (26, 55, 56). Considering that this study evaluated animals with skin or ear infections, the high prevalence found (76.00%) was expected. Interestingly, a high prevalence of SP in humans was found as well. Although cases of infections in humans have been reported (8, 16, 20, 57), the carrier condition attributed to humans is considered occasional. Like this study, in most reported cases, there was previous contact with dogs (8, 56, 58). The WGS analysis showed that in three cases, isolates from humans and their dogs were highly similar, suggesting transmission between hosts. Regarding the other sequenced samples, there was no evidence of transmission, and it was hypothesized that this could have occurred due to the acquisition of other strains shed by the animals, as dogs can eliminate SP through different routes such as the nose, pharynx, urogenital tract, rectum, and even via ocular discharge (58–61), or due the acquisition of a strain present in the environment, as the pathogen can persist in the environment for a long time (62).

The new MLST allelic profiles identified in the study show the occurrence of strains different from those deposited in pubMLST. The only known ST (1005) was associated with an SP isolated from a dog with otitis, originating from the state of Mato Grosso.^5^ We did not detect globally recognized MRSP STs such as ST71, which has been previously detected in Brazil (23, 60). Overall, the study indicates a broad genetic diversity of SP in the region.

The methicillin resistance (oxacillin) *in vitro* is a strong indicator of the presence of the *mecA* gene in SP, located on the mobile genetic element SCCmec (63). MRSP is recognized as a complicating bacterium in veterinary hospitals (64). The prevalence of MRSP in dogs with clinical infections varies widely, with reports ranging from 2 to 94.28% (14, 26, 65–67), and in the study, MRSP was identified in less than 50% of samples from dogs and their tutors. *In vitro* resistance to cefovecin, a third generation long-acting *β*-lactam antibiotic developed for use in dogs and cats (68), has also been correlated with the presence of *mecA* (69). Literature reports indicate good susceptibility of SP strains to cefovecin (69–71), as observed in our study. We observed a moderate or insignificant association between phenotypic resistance to oxacillin and cefovecin and the presence of the *mecA* gene, respectively. The resistance to these drugs may have occurred due to alternative mechanisms, such as β-lactamase production, as evidenced by the high prevalence of β-lactamase genes in the sequenced samples, similar to findings in previous studies (21, 24).

Although human infection by MRSP has been correlated with the presence of dogs in the household (9), the occurrence of MRSP in both dogs and their tutors simultaneously is underexplored. We found that dogs and their guardians exhibited similar percentages of MRSP, as described by Findik et al. (56), suggesting that contact with dogs is indeed a risk factor for humans becoming carriers of this bacterium. However, regarding the presence of the *mecA, mecA1,* and *mecA2* genes, the association between the simultaneous presence of these genes in a strain isolated from the dog and in its guardian was moderate or insignificant. We believe several factors may explain these results: (i) genetic exchange between strains did not actively occur; (ii) loss of resistance genes by bacteria during transmission from dog to tutor or vice versa; (iii) genotypic differences in persistently colonized SP isolates from dogs and tutors reflected in antimicrobial susceptibility (71). Adiguzel et al. (72), when analyzing 3,482 clinical isolates of methicillin-resistant Staphylococcus (MRS) in dogs and cats between 2012 and 2019, revealed a high prevalence of *S. pseudintermedius* and coagulase-negative *Staphylococcus* with frequent cross-resistance to antibiotics.

The use of antimicrobials is considered a risk factor for increasing SP resistance (73–75), a fact also observed by us for *β*-lactam antimicrobials. However, the association between antimicrobial use and resistance to oxacillin or cefovecin was strong or moderate, suggesting once again that other factors may be influencing this relationship.

Although SP has shown good susceptibility to gentamicin (66, 76–78), resistant strains have been detected (79, 80). We found a low percentage of resistance in isolation from dogs and tutors, and there was no association between MIC values among SP isolated from dogs and their tutors. Of the sequenced samples, 6/21 (%) exhibited at least one aminoglycoside resistance gene, but only one sample from a tutor showed phenotypic resistance. This may be related to different selection pressures, as even though the pathogen carries resistance genes, their expression is regulated by challenges imposed on the pathogen in the environment (81). Two other human samples exhibited phenotypic resistance without the presence of genes related to this class, suggesting that other mechanism(s) may have been responsible for the resistance. Although no gene showed a significant association with phenotypic resistance, the *aac(6′)-Ie/aph(2″)-Ia* gene is commonly associated with gentamicin resistance (82), unlike *ANT(6)-Ia/aad(6)* and *aph(3′)-IIIa*, which are typically linked to resistance to streptomycin and kanamycin/neomycin, respectively (83, 84).

Regarding resistance to macrolides, we found a low number of strains with the *msr(A)* gene. This gene encodes an ABC transporter and rRNA methylase that can confer resistance to streptogramin B and macrolide antibiotics (85, 86). However, of the sequenced samples, 7/21 (33.33%) exhibited at least one gene related to resistance to this class, indicating that these strains possess mechanisms that qualify them to resist drugs in this class. Of the samples sequenced, one guardian sample was positive for the *msrA* gene in the PCR, and negative after sequencing. This gene has already been found exclusively in plasmids (87), and we believe that this isolate may have lost a plasmid containing this gene, since the sample was reactivated at two different times: one for PCR and the other for DNA extraction to send for sequencing. Another plausible hypothesis is that the gene was fragmented across multiple contigs during sequencing, which may have impeded its detection by AMRfinderplus due to coverage-based filtering thresholds.

In addition to genes related to resistance to aminoglycosides, *β*-lactams, and macrolides, we identified genes conferring resistance to phenicols, streptothricin, tetracyclines, trimethoprim, and point mutations in genes capable of conferring resistance to quinolones. This finding demonstrates a diverse genetic repertoire in SP in Brazil, consistent with previous observations (24). The near-universal presence of the *blaIZR1* operon across the sequenced genomes has been reported in previous studies (12, 13) and suggests a potential for β-lactam resistance. However, phenotypic resistance was observed in only six isolates, and the lack of a strong genotype–phenotype association may be attributed to (1) differences in gene expression (88) and (2) polymorphisms within the *blaZ* gene, which may have deleterious effects on its function (89).

Additionally, a gene related to resistance to heavy metals (*qacG*) was found in genomes of SP isolated from dogs and tutors, suggesting that these strains have the capacity to resist disinfectants such as chlorhexidine digluconate (90), potentially becoming a persistent problem in veterinary environments or even within the household environment.

The process of conjugation between plasmids is a crucial mechanism of horizontal gene transfer (HGT), particularly significant for the spread of resistance genes (91). We have identified resistance genes associated with aminoglycosides, macrolides, phenicols, streptothricin and quarternary ammonium, especially in non-mobilizable plasmids, i.e., plasmids incapable of initiating conjugation themselves (47) that relies on transformation or transduction for propagation (92, 93). Overall, SP from both humans and dogs demonstrated the ability to exchange resistance genes to important antibiotics and biocides via HGT.

The absence of the PVL gene and the low prevalence of *fnbB* gene were unexpected, as these genes are important for establishing cytotoxic effects (2) and promoting adhesion to host cells (94, 95). Conversely, in the sequenced genomes, we detected the presence of virulent genes *sea, sec, seh,* and *sell*, related to five individuals and one animal sample. These toxins exhibit emetic and superantigenic activities in primate models, categorizing them as SEs (superantigens that cause food poisoning), with *sea, sec,* and *seh* genes indicating potential to cause foodborne illness (96). The *sea* and *sec* genes have been previously described in human samples, suggesting higher carriage rates in human isolates compared to animal and environmental isolates (97). These genes were also found in samples from both healthy and diseased animals, implying that these enterotoxins may not significantly contribute to disease pathogenesis in animals (98, 99).

Human contact with SP-positive dogs increases the likelihood of humans becoming a carrier of the bacteria by fivefold. However, sleeping with the dog increased the likelihood of the dog becoming SP-positive by sixfold compared to those that slept in their own bed. Our findings suggest that, depending on the tutor’s immunity and the colonization abilities of specific bacterial strains, the tutor may serve as a reservoir for the bacterium, potentially facilitating its transmission to the dog. Hanselman et al. (55) found that 50% of veterinarians were nasal carriers of SP, indicating that repeated contact with dogs may lead to carrier status. Additionally, dogs bathed in grooming facilities were four times more likely to be colonized by SP than those bathed at home, and we hypothesize that exposure to other dogs, the environment, and grooming professionals may promote SP colonization in these animals.

The diagnostic test provides values for susceptibility, specificity, positive predictive value, and negative predictive value, which are indicators of the viability of an analytical method. Comparing the results of disk diffusion tests, commonly used in most commercial laboratories, with those of MIC, considered the gold standard, can assist veterinarians, especially those dealing with small animals, in clinical practice. The susceptibility and specificity results showed that the disk diffusion method is not a good option for determining strains resistant to these antimicrobials. This method, while having good specificity, may result in a higher number of false negatives and classify resistant strains as susceptibility (48). In assessing antimicrobial resistance, it is ideal for tests to have high susceptibility so that the number of strains falsely classified as susceptibility is also low. Using an antimicrobial to which the bacterium is resistant will increase selective pressure for these resistant strains, leading to treatment failure or recurrences (74, 75).

## 5 Conclusion

There was a high prevalence of SP isolates in samples from dogs, tutors, and both concurrently, with contact with positive dogs being a risk factor for human colonization. The main risk factors for SP infection in dogs were sleeping with a positive tutor and frequenting grooming facilities. The isolates showed low rates of resistance to oxacillin and cefovecin, and the presence of the *mecA* gene was not the most important mechanism. Previous use of *β*-lactams moderately influenced strain resistance. The disk diffusion diagnostic method was not effective for identifying strains resistant to oxacillin and cefovecin.

Given the genetic variability, diversity of resistance genes and plasmids, and evidence of SP transmission between dogs and humans reported in this study, the results demonstrated that SP could pose a risk to humans, like other *Staphylococcus* species. Continuous surveillance is necessary to understand the bacterial population in animals and to minimize transmission and disease in both animals and humans.

## Data Availability

The datasets presented in this study can be found in online repositories. The names of the repository/repositories and accession number(s) can be found in the article/Supplementary material.

## Footnotes

^1^ https://github.com/tseemann/snp-dists

^2^ https://github.com/tseemann/mlst

^3^ https://pubmlst.org/bigsdb?db=pubmlst_spseudintermedius_seqdef

^4^ https://github.com/tseemann/abricate

^5^ https://pubmlst.org/bigsdb?page=info&db=pubmlst_spseudintermedius_isolates&id=1125
